# Supplementary figures and images for: Impact of vitamin D on glycemic control and microvascular complications in type 2 diabetes: A cross-sectional study
Source: PLoS One. 2025 May 28;20(5):e0324729. doi: 10.1371/journal.pone.0324729 (PMC12118829; doi:10.1371/journal.pone.0324729)

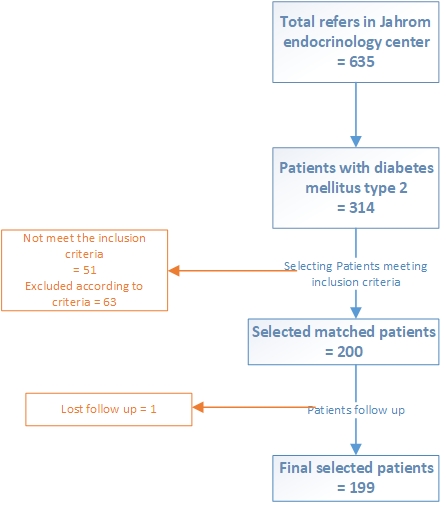

Supplement: S1 Fig — (JPG) [file pone.0324729.s001.jpg]

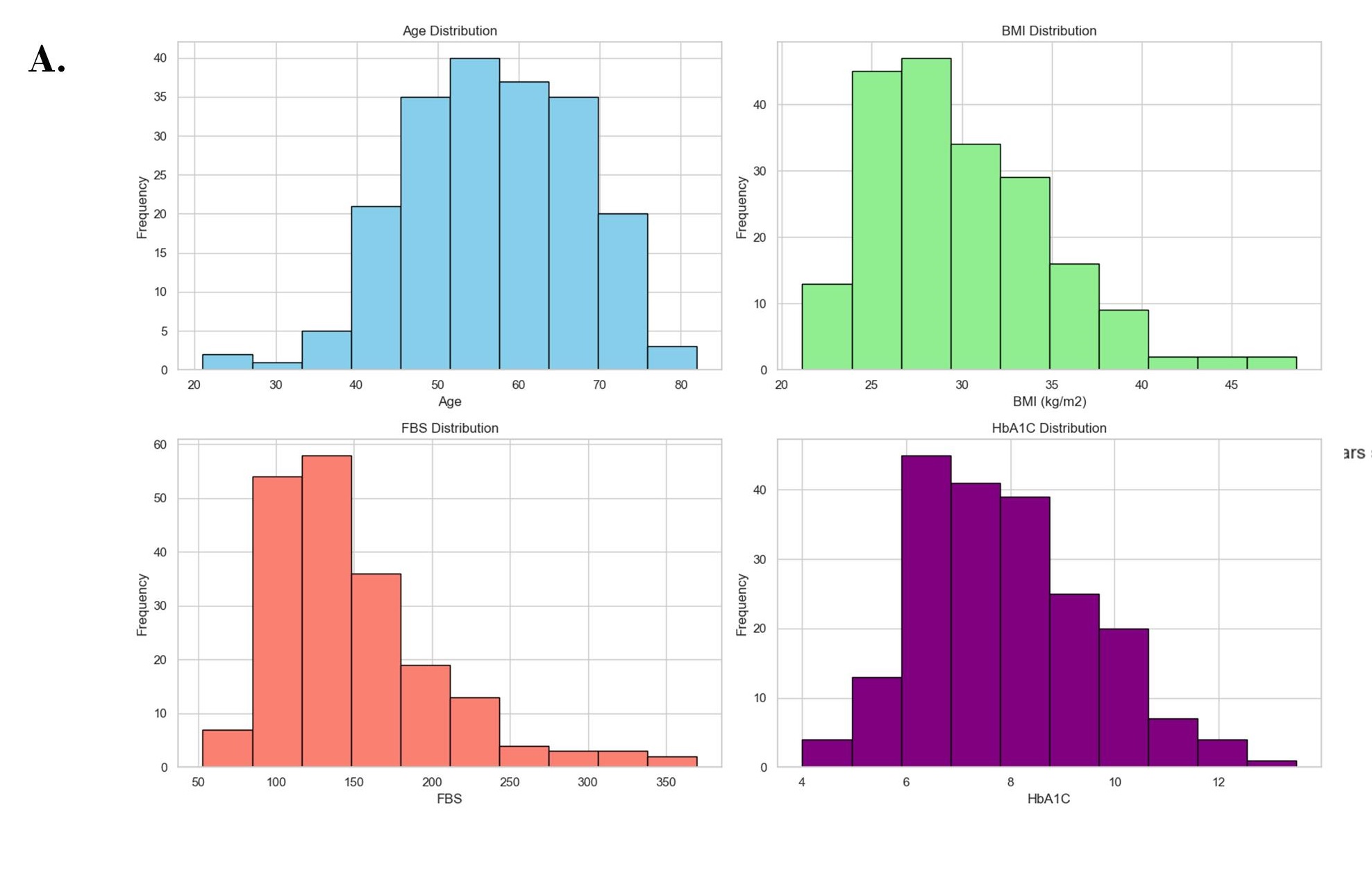

Supplement: S2A Fig — Age Distribution: Displays the frequency of participants by age (mean age = 56.79 ± 10.8 years). BMI Distribution: Shows the frequency of participants by body mass index (mean BMI = 28.91 kg/m²). FBS (Fasting Blood Sugar) Distribution: Depicts the frequency of participants based on their fasting blood sugar levels. HbA1C Distribution: Illustrates the frequency of participants by HbA1C percentage, an indicator of glycemic control. (JPG) [file pone.0324729.s002.jpg]

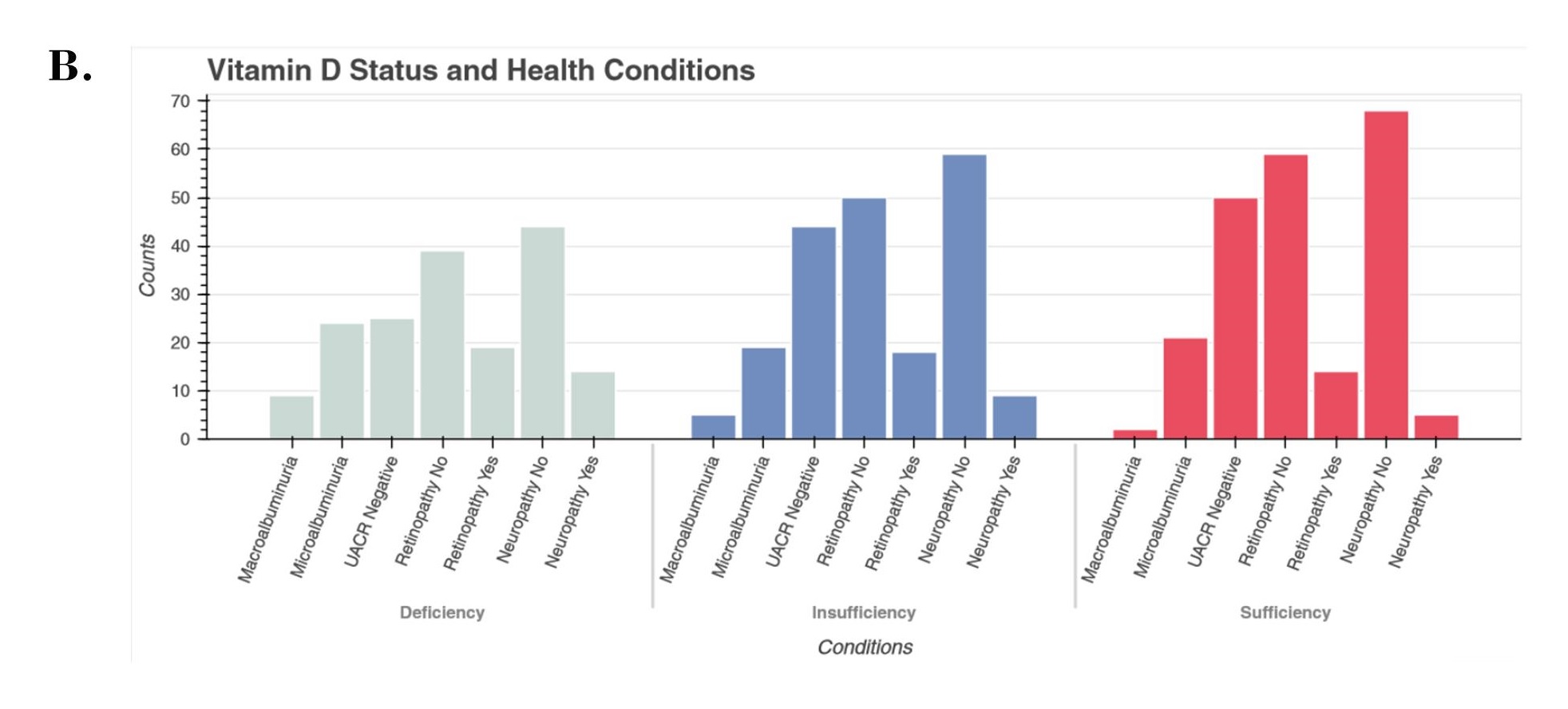

Supplement: S2B Fig — This bar chart shows the counts of patients categorized by vitamin D status (deficiency, insufficiency, and sufficiency) and the presence or absence of specific health conditions, including macroalbuminuria, retinopathy, and neuropathy. Deficiency, insufficiency, and sufficiency are compared for each health condition to highlight the relationship between vitamin D status and the prevalence of microvascular complications. (JPG) [file pone.0324729.s003.jpg]

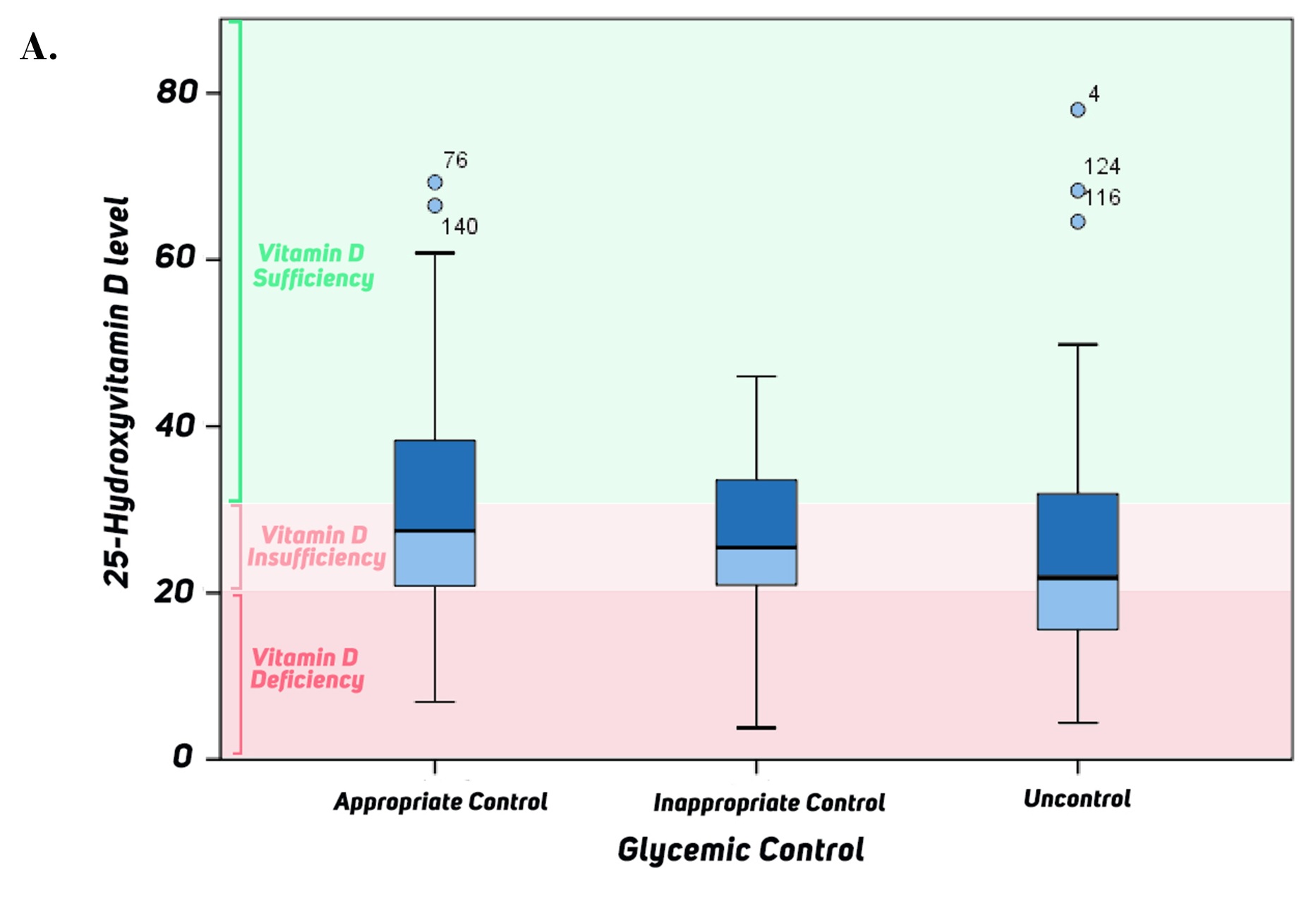

Supplement: S3A Fig — The Kruskal-Wallis test was used for statistical analysis. *p < 0.05 for comparisons between appropriately controlled vs. inappropriately controlled and vs. uncontrolled groups. Though significant, p-values are not shown in the figure. (JPG) [file pone.0324729.s004.jpg]

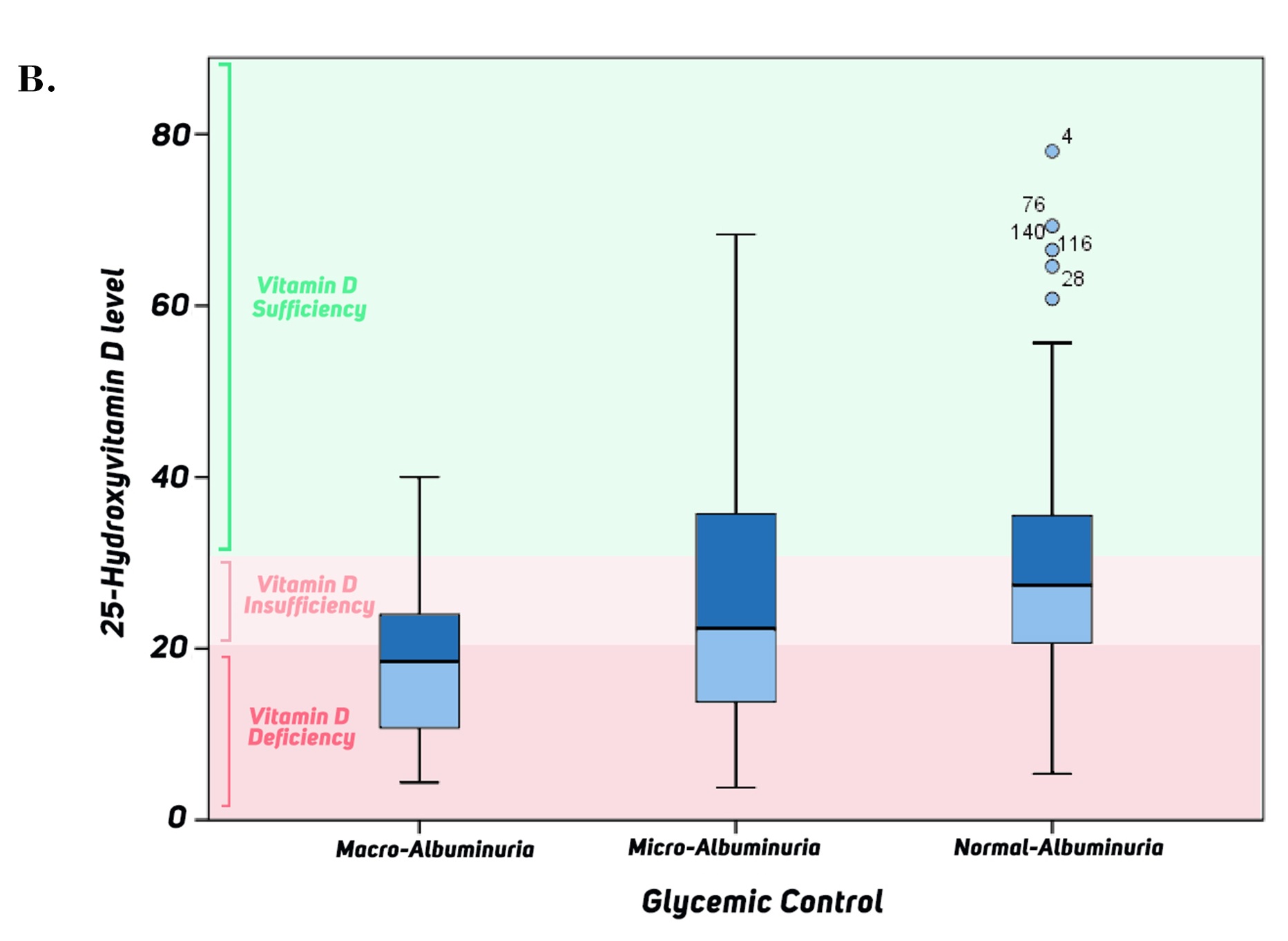

Supplement: S3B Fig — The Kruskal-Wallis test was used for statistical analysis. *p < 0.05 for comparisons between normoalbuminuria vs. microalbuminuria and macroalbuminuria. Although significant, p-values are not displayed in the figure. (JPG) [file pone.0324729.s005.jpg]
