# Supplementary material for: Impact of vitamin D on glycemic control and microvascular complications in type 2 diabetes: A cross-sectional study
Source: PLoS One. 2025 May 28;20(5):e0324729. doi: 10.1371/journal.pone.0324729 (PMC12118829; doi:10.1371/journal.pone.0324729)
Supplement: Attachment — Submitted filename: response to initial review.pdf [file pone.0324729.s007.pdf]

Dear **Editorial Team**,

Thank you for your detailed feedback regarding our manuscript submission. We appreciate your efforts in reviewing the manuscript, and we have made the following revisions based on your comments:

1. **Recruitment Period**: We have updated the Methods section to include the specific dates of the recruitment period, as follows: *This analytical cross-sectional study (started: September 16, 2021, and ended: March 23, 2022) includes 199 type-2 diabetic patients who were referred to the endocrinology clinic of Jahrom city.*
2. **Text Overlap**: We have carefully revised the manuscript to address the significant text overlap identified with previously published works. The duplicated text has been rephrased, and all sources have been properly cited. We are aware of the importance of avoiding word-for-word repetition, especially outside of the methods section, and we have ensured that the manuscript no longer contains any such issues. All citations have been fully updated and appropriately placed.

We believe these changes have improved the manuscript, and we are confident that the revisions now align with the submission guidelines. Please let us know if any further adjustments are needed. We look forward to your feedback upon resubmission.

Kind regards,
Salma Ahi, MD
