# Supplementary material for: Impact of vitamin D on glycemic control and microvascular complications in type 2 diabetes: A cross-sectional study
Source: PLoS One. 2025 May 28;20(5):e0324729. doi: 10.1371/journal.pone.0324729 (PMC12118829; doi:10.1371/journal.pone.0324729)
Supplement: Attachment — Submitted filename: Reports.docx [file pone.0324729.s006.docx]

Reviewer 1:

Minor revision

The author could comment on the burden of type 2 Diabetes mellitus in the study area.
The author could mention the status of Vitamin D Deficiency in the study area.
Methods 2nd paragraph can include exclusion criteria as the subheading.
If the BP was high, the author could mention whether a single reading or a series of readings were taken and the average noted down.
Was there any sample size calculation done? Or is any minimum sample size required calculated?
The author could mention if only newly diagnosed T2DM patients or patients with existing T2DM were included in the inclusion criteria.

Reviewer 2:

Minor revision

Please mention what was the basis for the sample size calculation.
What were the food habits of the study participants? Veg/non-veg/Vegan??
Discussion needs more recent article citation and literature description.
