# Supplementary material for: Impact of vitamin D on glycemic control and microvascular complications in type 2 diabetes: A cross-sectional study
Source: PLoS One. 2025 May 28;20(5):e0324729. doi: 10.1371/journal.pone.0324729 (PMC12118829; doi:10.1371/journal.pone.0324729)
Supplement: Attachment — Submitted filename: rebuttal letter.docx [file pone.0324729.s008.docx]

# Response to Editor

### Dear Editorial board of "PLOS ONE"

Following your letter regarding the manuscript " Impact of vitamin D on glycemic control and microvascular complications in type 2 diabetes: A cross-sectional study" submitted to the PLOS ONE for publication, we are sending the rebuttal letter explaining the modifications applied on the manuscript. We have implemented required revisions to the manuscript and we believe that the current revised paper complies with the referee’s constructive remarks. Besides addressing the critiques of the reviewers, we have evaluated the manuscript to add the suggested discussions by the reviewers. We found the excellent comments of the editor and reviewer very constructive and insightful, and we appreciate the time that you and the reviewer have devoted to assess our work. Therefore, all the helpful suggestions and comments have been fully considered. We appreciate the respectful editorial board and the reviewers for devoting their time and providing constructive and thoughtful critiques towards our primary manuscript. The revisions, starting with the last submission, are addressed below.

**The responses to the comments of the editor and reviewer can be found as follows.**

Journal Requirements:

When submitting your revision, we need you to address these additional requirements.

Comment 1. Please ensure that your manuscript meets PLOS ONE's style requirements, including those for file naming. The PLOS ONE style templates can be found at

<https://journals.plos.org/plosone/s/file?id=wjVg/PLOSOne_formatting_sample_main_body.pdf> and 
<https://journals.plos.org/plosone/s/file?id=ba62/PLOSOne_formatting_sample_title_authors_affiliations.pdf>

Author response - 1: We sincerely appreciate your valuable feedback. In response to your comments, we have carefully revised the manuscript to meet PLOS ONE's guidelines. The following changes have been made:

- The **title format** has been adjusted to comply with PLOS ONE standards.
- **Author names and affiliations** have been updated to the requested format.
- **Section headings** have been modified to align with the journal’s preferred style.
- **Figure legends and tables** have been repositioned within the text, placed after their first reference, as per PLOS ONE guidelines.
- The **abstract** has been revised to meet the journal’s requirements.

Comment 2. We noticed you have some minor occurrence of overlapping text with the following previous publication(s), which needs to be addressed:

- Microvascular Complications and Foot Care: Standards of Medical Care in Diabetes—2021 - <https://doi.org/10.2337/dc21-S011>
- Ultraviolet Radiation: A Hazard to Children and Adolescents  - <https://doi.org/10.1542/peds.2010-3502>

In your revision ensure you cite all your sources (including your own works), and quote or rephrase any duplicated text outside the methods section. Further consideration is dependent on these concerns being addressed.

Author response - 2: We sincerely appreciate your careful attention to this matter. We have thoroughly reviewed the manuscript and addressed the instances of overlapping text with the cited publications. The following corrective actions have been taken:

1. **Proper Citation**: All relevant sources, including the two referenced publications, have been appropriately cited where necessary.
2. **Rephrasing**: Any duplicated text outside the Methods section has been either rephrased for originality or properly quoted with attribution.
3. **Originality Check**: We have ensured that all remaining content is presented in our own words, with proper citations where prior work is referenced.

Comment 3. We note that you have indicated that there are restrictions to data sharing for this study. For studies involving human research participant data or other sensitive data, we encourage authors to share de-identified or anonymized data. However, when data cannot be publicly shared for ethical reasons, we allow authors to make their data sets available upon request. For information on unacceptable data access restrictions, please see <http://journals.plos.org/plosone/s/data-availability#loc-unacceptable-data-access-restrictions>.

Before we proceed with your manuscript, please address the following prompts:

a) If there are ethical or legal restrictions on sharing a de-identified data set, please explain them in detail (e.g., data contain potentially identifying or sensitive patient information, data are owned by a third-party organization, etc.) and who has imposed them (e.g., a Research Ethics Committee or Institutional Review Board, etc.). Please also provide contact information for a data access committee, ethics committee, or other institutional body to which data requests may be sent.

b) If there are no restrictions, please upload the minimal anonymized data set necessary to replicate your study findings to a stable, public repository and provide us with the relevant URLs, DOIs, or accession numbers. Please see <http://www.bmj.com/content/340/bmj.c181.long> for guidelines on how to de-identify and prepare clinical data for publication. For a list of recommended repositories, please see <https://journals.plos.org/plosone/s/recommended-repositories>. You also have the option of uploading the data as Supporting Information files, but we would recommend depositing data directly to a data repository if possible.

Please update your Data Availability statement in the submission form accordingly.

Author response - 3: We appreciate the journal’s policy on data sharing and fully support transparency in research. However, due to the sensitive nature of the data in this study, we are unable to publicly share the dataset for the following reasons:

1. **Patient Privacy Concerns**: Even after de-identification, the data contain potentially identifiable patient information, making unrestricted sharing ethically problematic.
2. **Ethical Restrictions**: The **Ethics Committee of Jahrom University of Medical Sciences** has imposed restrictions on public data sharing to protect participant confidentiality.

**Data Access Process**:

While the data cannot be made publicly available, qualified researchers may request access through a formal application to: Jahrom University of Medical Science (**info@jums.ac.ir**)

We have updated the **Data Availability Statement** in the manuscript to reflect these restrictions and provide clear instructions for data requests.

Thank you for your understanding. Please let us know if further clarification is needed.

Comment 4. In this instance it seems there may be acceptable restrictions in place that prevent the public sharing of your minimal data. However, in line with our goal of ensuring long-term data availability to all interested researchers, PLOS’ Data Policy states that authors cannot be the sole named individuals responsible for ensuring data access (<http://journals.plos.org/plosone/s/data-availability#loc-acceptable-data-sharing-methods>).

Data requests to a non-author institutional point of contact, such as a data access or ethics committee, helps guarantee long term stability and availability of data. Providing interested researchers with a durable point of contact ensures data will be accessible even if an author changes email addresses, institutions, or becomes unavailable to answer requests.

Before we proceed with your manuscript, please also provide non-author contact information (phone/email/hyperlink) for a data access committee, ethics committee, or other institutional body to which data requests may be sent. If no institutional body is available to respond to requests for your minimal data, please consider if there any institutional representatives who did not collaborate in the study, and are not listed as authors on the manuscript, who would be able to hold the data and respond to external requests for data access? If so, please provide their contact information (i.e., email address). Please also provide details on how you will ensure persistent or long-term data storage and availability.

Author Response - 4: Thank you for your guidance. We have updated our data availability statement and provide the following non-author institutional contact and details for long-term data access:

**Revised Data Availability Statement:**
"Due to ethical restrictions imposed by the Ethics Committee of Jahrom University of Medical Sciences to protect participant confidentiality, the data underlying this study cannot be made publicly available. Qualified researchers may request access to the de-identified minimal dataset by contacting the university's independent Ethics Committee at [info@jums.ac.ir](mailto:info@jums.ac.ir) (with email title: **Access to research data**) or +98 (715) 4474992. Requests will be reviewed for compliance with ethical standards and institutional regulations. The data will be stored securely in the university’s institutional repository, which guarantees long-term preservation and accessibility for approved researchers, irrespective of author availability."

Comment 5. PLOS requires an ORCID iD for the corresponding author in Editorial Manager on papers submitted after December 6th, 2016. Please ensure that you have an ORCID iD and that it is validated in Editorial Manager. To do this, go to ‘Update my Information’ (in the upper left-hand corner of the main menu), and click on the Fetch/Validate link next to the ORCID field. This will take you to the ORCID site and allow you to create a new iD or authenticate a pre-existing iD in Editorial Manager.

Author Response - 5: Thank you for your comment. We confirm that the corresponding author’s ORCID iD has been validated in Editorial Manager as required. The steps outlined were followed to ensure compliance:

1. The corresponding author’s ORCID iD (**0000-0001-9622-2895**) has been updated in the Editorial Manager system.
2. The ORCID record is now fully linked and updated in the submission profile.

We have ensured all requirements are met and appreciate your guidance.

Comment 6. We note you have included a table to which you do not refer in the text of your manuscript. Please ensure that you refer to Table 3 in your text; if accepted, production will need this reference to link the reader to the Table.

Author Response - 6: Thank you for highlighting this oversight. We confirm that **Table 3** is now referenced in the final paragraph of the **Results** section. The manuscript has been updated accordingly.

Comment 7. Please include captions for your Supporting Information files at the end of your manuscript, and update any in-text citations to match accordingly. Please see our Supporting Information guidelines for more information: <http://journals.plos.org/plosone/s/supporting-information>.

Author Response – 7: Thank you for your feedback. We have added captions for the Supporting Information file at the end of the manuscript and updated all in-text citations to reference these materials appropriately.

Comment 8. Please review your reference list to ensure that it is complete and correct. If you have cited papers that have been retracted, please include the rationale for doing so in the manuscript text, or remove these references and replace them with relevant current references. Any changes to the reference list should be mentioned in the rebuttal letter that accompanies your revised manuscript. If you need to cite a retracted article, indicate the article’s retracted status in the References list and also include a citation and full reference for the retraction notice.

Author Response – 8: Thank you for your valuable comment. We have carefully reviewed the reference list to ensure its completeness and accuracy. We confirm that none of the cited papers in our manuscript have been retracted. All references are current and relevant to the study. Also, the reference list has been verified for proper formatting, completeness, and alignment with in-text citations. No changes were required.

# Peer reviewers’ comments and Authors′ responses

Reviewers' comments:

Reviewer's Responses to Questions

**Comments to the Author**

1. Is the manuscript technically sound, and do the data support the conclusions?

The manuscript must describe a technically sound piece of scientific research with data that supports the conclusions. Experiments must have been conducted rigorously, with appropriate controls, replication, and sample sizes. The conclusions must be drawn appropriately based on the data presented.

Reviewer #1: Yes

Reviewer #2: Yes

2. Has the statistical analysis been performed appropriately and rigorously?

Reviewer #1: Yes

Reviewer #2: Yes

3. Have the authors made all data underlying the findings in their manuscript fully available?

The [PLOS Data policy](http://track.editorialmanager.com/CL0/http:%2F%2Fwww.plosone.org%2Fstatic%2Fpolicies.action%23sharing/1/010f019544b72d5f-56d5a9f1-e1b1-4424-acf6-7aae17de7f78-000000/w1_sGOH5Vis2cPV4YJC7_cbEIbNFfmxP45Hoq83rO0w=200) requires authors to make all data underlying the findings described in their manuscript fully available without restriction, with rare exception (please refer to the Data Availability Statement in the manuscript PDF file). The data should be provided as part of the manuscript or its supporting information, or deposited to a public repository. For example, in addition to summary statistics, the data points behind means, medians and variance measures should be available. If there are restrictions on publicly sharing data—e.g. participant privacy or use of data from a third party—those must be specified.

Reviewer #1: No

Reviewer #2: Yes

4. Is the manuscript presented in an intelligible fashion and written in standard English?

PLOS ONE does not copyedit accepted manuscripts, so the language in submitted articles must be clear, correct, and unambiguous. Any typographical or grammatical errors should be corrected at revision, so please note any specific errors here.

Reviewer #1: Yes

Reviewer #2: Yes

Review Comments to the Author

Please use the space provided to explain your answers to the questions above. You may also include additional comments for the author, including concerns about dual publication, research ethics, or publication ethics. (Please upload your review as an attachment if it exceeds 20,000 characters)

Reviewer #1:

Author: We sincerely appreciate your insightful feedback and constructive comments, which have been invaluable in enhancing the quality of our manuscript. Your suggestions have significantly strengthened the clarity, rigor, and presentation of our work. We have carefully addressed each of your points.

R1-comment-1: The author could comment on the burden of type 2 Diabetes mellitus in the study area.

Author-Response-1: Thank you for your valuable comment. We have added a paragraph addressing this point (Paragraph 3 of the Introduction).

R1-comment-2: The author could mention the status of Vitamin D Deficiency in the study area.

Author-Response-2: This point has been addressed in paragraph 4 of the introduction. Thank you for your comment.

R1-comment-3: Methods 2nd paragraph can include exclusion criteria as the subheading.

Author-Response-3: Many thanks for noting this issue, we added exclusion criteria as a subheading in the 2^nd^ paragraph of methods.

R1-comment-4: If the BP was high, the author could mention whether a single reading or a series of readings were taken and the average noted down.

Author-Response-4: Thank you for raising this important point. In our study, blood pressure (BP) measurements were performed in accordance with the latest international protocols. For patients with elevated BP, two or more readings were taken at 1–2-minute intervals after the initial 10-minute rest period, and the average of these readings was recorded. This approach aligns with the 2023 World Health Organization(1) (WHO) and American Heart Association(2) (AHA) guidelines, which emphasize the importance of multiple BP measurements to confirm hypertension and reduce variability.

For hypertensive patients or those with high initial readings, according to current guidelines, we rechecked the device and cuff size and also patients’ proper positioning. In addition we asked the patient for out-of-office checking the BP if feasible or another session BP measurement in the office.

We have clarified this methodology in the revised manuscript (Methods section, paragraph 3) to reflect these details. Thank you for your valuable feedback.

R1-comment-5: Was there any sample size calculation done? Or is any minimum sample size required calculated?

Author-Response-5: (also see response to reviewer 2 – comment 1)Thank you for your critical question regarding sample size determination. Below is a detailed statistical description based on the study’s results and methodology:

1. Sample Size Rationale:

The study included 199 participants, which was determined by feasibility (availability of eligible patients during the study period). While no formal a priori power calculation was conducted, we performed **post-hoc power analyses** to assess the adequacy of the sample size for detecting the observed effects.

2. Post-Hoc Power Analysis:

Using the observed effect sizes from the results:

**Neuropathy**: Large effect size (η = 0.975, *p* < 0.001).

With *N* = 199, power exceeds **99%** (α = 0.05) for detecting this strong association.

**HbA1C and Vitamin D correlation**: Small-to-moderate inverse correlation (*r* = -0.19).

Power ≈ **80%** to detect this effect (*N* = 199, α = 0.05, two-tailed).

**UACR and Vitamin D correlation**: Weak inverse correlation (*r* = -0.175).

Power ≈ **70%** (*N* = 199, α = 0.05).

3. Key Observations

**Detectable effects**: The sample size was sufficient to identify **clinically meaningful associations** (e.g., neuropathy’s strong link to vitamin D deficiency) but may lack power for smaller effects (e.g., weaker correlations like UACR).

**Group comparisons**: For subgroup analyses (e.g., retinopathy [*n* = 51], neuropathy [*n* = 28]), power decreases due to smaller group sizes. For example:

Retinopathy: Mean vitamin D difference = **3.37 ng/ml** (SD ≈ 13.7).

Cohen’s *d* = 0.25 → Power ≈ **40%** (underpowered for small effects).

4. Limitations:

**No formal a priori calculation**: This is a limitation, as highlighted in the revised manuscript.

**Retrospective power**: While post-hoc analyses suggest adequate power for primary findings (e.g., neuropathy, HbA1C), smaller effects (e.g., retinopathy) require cautious interpretation.

**Future studies**: Larger cohorts or targeted recruitment for subgroups (e.g., macroalbuminuria [*n* = 16]) are needed to confirm associations with smaller effect sizes.

5. Revised Manuscript Update

We added the following to the **Limitations section**:
*"The sample size was determined by feasibility, and no formal a priori power calculation was conducted. Post-hoc analyses indicated sufficient power (≥80%) for detecting moderate-to-large effects (e.g., neuropathy-HbA1C correlations) but limited power for smaller associations (e.g., retinopathy). Future studies should prioritize prospective power calculations to validate these findings."*

R1-comment-6: The author could mention if only newly diagnosed T2DM patients or patients with existing T2DM were included in the inclusion criteria.

Author-Response-6: Thank you for this important clarification. In our study, **only patients with established (previously diagnosed) type 2 diabetes mellitus (T2DM)** were included. Newly diagnosed T2DM cases were excluded based on the following rationale:

1. **Requirement for Longitudinal Clinical Data collection**:
   Microvascular complications (e.g., nephropathy, retinopathy, neuropathy) develop over years of diabetes progression. By focusing on established T2DM patients, we ensured sufficient time had elapsed for complications to manifest, and of course to be diagnosed by the physician and documented in their medical records (for example past history of admission to hospital due to diabetic foot)
2. **Need for Multiple Visits and Lab Assessments**:
   Complications like nephropathy (requiring repeated UACR measurements) and retinopathy (confirmed via ophthalmologist evaluations) demand longitudinal follow-up and repeated testing. Newly diagnosed patients often lack this historical data, making them unsuitable for assessing chronic complications.
3. **Comprehensive Medical Records**:
   Established patients had extensive prior records (e.g., HbA1C trends, medication history, duration of diabetes), which are critical for analyzing relationships between long-term glycemic control, vitamin D levels, and complications.
4. **Homogeneity of the Cohort**:
   Including only established T2DM patients minimized variability in disease duration and treatment regimens, reducing confounding factors that could arise with newly diagnosed cases (e.g., acute metabolic adjustments).

**Revised Manuscript Clarification (The 1^st^ paragraph of methods):**
*"Participants were required to have a confirmed diagnosis of T2DM for at least 1 year prior to enrollment. This criterion ensured access to longitudinal clinical data, including prior HbA1C measurements, complication screenings, and treatment histories, which were essential for analyzing chronic microvascular outcomes."*

Reviewer #2:

R2-comment-1: Please mention what was the basis for the sample size calculation.

Author-Response-1: (Also see response to reviewer 2 – comment 5) Thank you for your valuable comment regarding our sample size determination. We have revised our explanation to provide a more detailed rationale, supported by well-established references and real studies in this field.

1. **Basis for Sample Size**

Our study’s sample size (N = 199) was primarily determined by feasibility considerations that are common in observational studies of chronic diseases such as type 2 diabetes (T2DM). Key factors included:

- **Resource and Logistical Constraints:**
  The study design required multiple clinical visits, specialist assessments (e.g., ophthalmologic evaluations), and laboratory tests (e.g., urine albumin-to-creatinine ratio [UACR], HbA1C, serum vitamin D), which limited the number of participants available during the recruitment period.
- **Eligibility Criteria:**
  Restricting participation to patients with established T2DM ensured the availability of sufficient historical clinical data for a robust assessment of diabetic complications.
- **Subgroup Analyses:**
  To enable meaningful subgroup analyses—such as comparing patients with neuropathy (n ≈ 28) versus retinopathy (n ≈ 51)—we targeted a minimum of approximately 50 participants per subgroup. This approach is consistent with recommendations in sample size literature (e.g., Machin et al., 2009(3)) and parallels methodologies employed in similar studies [e.g., Zoppini et al., 2015(4); Scragg et al., 2004(5)].

2. **Validation of Sample Size Against Findings**

Post-hoc power analyses confirmed that our sample size was sufficient to detect clinically relevant associations:

- **Neuropathy:**
  We observed a strong association (η = 0.975, p < 0.001) with statistical power exceeding 80%.
- **HbA1C and Vitamin D:**
  A moderate inverse correlation (r = -0.19, p = 0.007) was significant, while a slightly weaker association (e.g., UACR-vitamin D, r = -0.175) achieved an estimated power of ~70%, which is acceptable for an exploratory study.

These findings align with previous research such as that by Chiu et al. (6) and Pittas et al. (7), which documented significant links between vitamin D levels and various diabetic complications.

3. **Methodological Precedents in the Field**

Our feasibility-driven approach is well supported in the literature:

- **Feasibility and Resource Constraints:**
  As described by Hulley et al.(8) and Machin et al.(3), observational studies in chronic disease often use feasibility as a key determinant for sample size due to the complex logistics of collecting extensive clinical data.
- **Real-World Examples:**
  - **Zoppini et al.(****4****):** Investigated the association between vitamin D status and albuminuria in T2DM patients, where the sample size was largely determined by the availability of eligible patients.

4. **Manuscript Update**

We added the following to the **Limitations section**:
*"The sample size was determined by feasibility, and no formal a priori power calculation was conducted. Post-hoc analyses indicated sufficient power (≥80%) for detecting moderate-to-large effects (e.g., neuropathy-HbA1C correlations) but limited power for smaller associations (e.g., retinopathy). Future studies should prioritize prospective power calculations to validate these findings."*

R2-comment-2: What were the food habits of the study participants? Veg/non-veg/Vegan??

Author-Response-2: Thank you for your comment regarding the dietary habits of our participants. We systematically recorded food habits using a dietary part of questionnaire at baseline. Our findings indicated that the participants predominantly followed typical Iranian dietary patterns, which feature a balanced mix of plant-based foods and animal proteins. We did not specifically focus on or recruit vegetarians, vegans, or individuals with restrictive dietary practices. Instead, our inclusion criteria ensured that all participants had diets consistent with common regional Iranian food habits. This approach allowed us to maintain dietary homogeneity and minimize confounding effects from markedly different nutritional practices. Also we mention this moot point in the article context (The 1^st^ paragraph of methods).

R2-comment-3: Discussion needs more recent article citation and literature description.

Author-Response-3: We appreciate your suggestion for incorporating more recent citations and an enriched literature discussion. In response, we have updated the discussion to include two new paragraphs that integrate findings from very recent studies, also we added several parts to the discussion to address literature review in more details and more recent studies.

In these additions, we compare our observations with a cross‐sectional study by Chen et al. (2022), which corroborated that lower serum 25(OH)D levels are significantly associated with a higher prevalence of microvascular complications (including retinopathy and nephropathy) in type 2 diabetes patients, even after adjusting for glycemic control. This study’s emphasis on regional and dietary influences on vitamin D status particularly supports our findings in a cohort following typical Iranian dietary habits.

Furthermore, we have incorporated insights from a systematic review and meta-analysis by Xuan et al. (2022)(9), which evaluated vitamin D supplementation in diabetic nephropathy. Their analysis of 10 randomized controlled trials involving 651 patients revealed that vitamin D supplementation significantly increased serum vitamin D levels while reducing urinary protein excretion and blood creatinine levels. These outcomes underscore the protective role of vitamin D in mitigating kidney dysfunction and align with our observation of better glycemic control and lower complication rates in patients with higher serum 25(OH)D levels. Together, these recent studies not only validate our results but also highlight the potential therapeutic benefits of maintaining adequate vitamin D status in the management of type 2 diabetes complications.

1.	Al-Makki A, DiPette D, Whelton PK, Murad MH, Mustafa RA, Acharya S, et al. Hypertension pharmacological treatment in adults: a World Health Organization guideline executive summary. Hypertension. 2022;79(1):293-301.

2.	Whelton PK, Carey RM, Mancia G, Kreutz R, Bundy JD, Williams B. Harmonization of the American College of Cardiology/American Heart Association and European Society of Cardiology/European Society of Hypertension blood pressure/hypertension guidelines: comparisons, reflections, and recommendations. European heart journal. 2022;43(35):3302-11.

3.	Machin D, Campbell MJ, Tan SB, Tan SH. Sample size tables for clinical studies: John Wiley & Sons; 2011.

4.	Zoppini G, Galletti A, Targher G, Brangani C, Pichiri I, Trombetta M, et al. Lower levels of 25-hydroxyvitamin D3 are associated with a higher prevalence of microvascular complications in patients with type 2 diabetes. BMJ Open Diabetes Research and Care. 2015;3(1):e000058.

5.	Scragg R, Sowers M, Bell C. Serum 25-hydroxyvitamin D, diabetes, and ethnicity in the Third National Health and Nutrition Examination Survey. Diabetes care. 2004;27(12):2813-8.

6.	Chiu KC, Chu A, Go VLW, Saad MF. Hypovitaminosis D is associated with insulin resistance and β cell dysfunction. The American journal of clinical nutrition. 2004;79(5):820-5.

7.	Pittas AG, Lau J, Hu FB, Dawson-Hughes B. The role of vitamin D and calcium in type 2 diabetes. A systematic review and meta-analysis. The Journal of Clinical Endocrinology & Metabolism. 2007;92(6):2017-29.

8.	Hulley S, Cummings S, Browner W, Grady D, Newman T. Designing clinical research fourth edition. Lippincott Williams & Wilkins, Philadelphia2013. p. 39.

9.	Xuan S, Jin Z, Zhe W, Huai-en B, Chun-ying T, Dong-jun W, et al. A systematic review and meta-analysis of randomized control trials of vitamin D supplementation in diabetic nephropathy. International Journal of Diabetes in Developing Countries. 2023;43(1):4-11.
